# The molecular basis of the dichotomous functionality of MAP4K4 in proliferation and cell motility control in cancer

**DOI:** 10.3389/fonc.2022.1059513

**Published:** 2022-12-08

**Authors:** Dejana Jovanovic, Shen Yan, Martin Baumgartner

**Affiliations:** Pediatric Molecular Neuro-Oncology Research, Children’s Research Centre, Division of Oncology, University Children’s Hospital Zürich, Zürich, Switzerland

**Keywords:** MAP4K4, proliferation, motility, STRIPAK complex, actin dynamics, PKC delta, VASP

## Abstract

The finely tuned integration of intra- and extracellular cues by components of the mitogen-activated protein kinase (MAPK) signaling pathways controls the mutually exclusive phenotypic manifestations of uncontrolled growth and tumor cell dissemination. The Ser/Thr kinase MAP4K4 is an upstream integrator of extracellular cues involved in both proliferation and cell motility control. Initially identified as an activator of the c-Jun N-terminal kinase (JNK), the discovery of diverse functions and additional effectors of MAP4K4 beyond JNK signaling has considerably broadened our understanding of this complex kinase. The implication of MAP4K4 in the regulation of cytoskeleton dynamics and cell motility provided essential insights into its role as a pro-metastatic kinase in cancer. However, the more recently revealed role of MAP4K4 as an activator of the Hippo tumor suppressor pathway has complicated the understanding of MAP4K4 as an oncogenic driver kinase. To develop a better understanding of the diverse functions of MAP4K4 and their potential significance in oncogenesis and tumor progression, we have collected and assessed the current evidence of MAP4K4 implication in molecular mechanisms that control proliferation and promote cell motility. A better understanding of these mechanisms is particularly relevant in the brain, where MAP4K4 is highly expressed and under pathological conditions either drives neuronal cell death in neurodegenerative diseases or cell dissemination in malignant tumors. We review established effectors and present novel interactors of MAP4K4, which offer mechanistic insights into MAP4K4 function and may inspire novel intervention strategies. We discuss possible implications of novel interactors in tumor growth and dissemination and evaluate potential therapeutic strategies to selectively repress pro-oncogenic functions of MAP4K4.

## 1 Introduction

Since the molecular cloning of the human ortholog HGK/MAP4K4 (1) (further referred to as MAP4K4) of the mouse Nck-interacting kinase and the *Caenorhabditis elegans* MIG-15 (2) in 2003, more than 200 studies have explored the molecular interactors and the biological functions of this kinase. In these studies, three main functional axes have been identified for MAP4K4: The regulation of insulin signaling and glucose up-take, the control of actin cytoskeleton dynamics and the regulation of endothelial integrity. De-regulation of these core functions contributes to various pathologies at organism level, of which increased MAP4K4 was mostly associated with tumors derived from epithelial tissues. As for many emerging kinase targets, small molecule inhibitors of MAP4K4 have been developed and their anti-cancer activities explored. However, only for one of these inhibitors a clinical phase 1 study was opened recently. One possible reason for the failure of some of these compounds may be the toxicity associated with MAP4K4 inhibition in tissues with high physiological MAP4K4 expression. The lack of an effective, non-toxic interference strategy to repress pathogenic functions of MAP4K4 specifically is thus one bottleneck of therapy development for pathologies caused by aberrant MAP4K4 function. Another remarkable finding in the cancer field is the anti-proliferative activity of MAP4K4 through the activation of Hippo tumor suppressor signaling (3–5). This questions the potential of direct MAP4K4 inhibition as a strategy to repress tumor dissemination, as it may lead to increased proliferation due to the shut-down of Hippo signaling.

In this review, we first provide an overview of MAP4K4 functions across tissues. We then more specifically discuss MAP4K4 functions associated with tumor growth and progression in the context of its dichotomous functionality as a promotor of invasion and repressor of tumor cell proliferation. We present a collection of upstream regulators and downstream effectors of MAP4K4, which constitute potential targets for therapeutic interventions to prevent toxicities associated with direct targeting of MAP4K4. Latter is of particular relevance in the context of the association of high MAP4K4 expression with the invasive behavior of tumors spreading in the central nervous system (CNS) - such as the most common malignant pediatric brain tumor medulloblastoma (6), or glioblastoma (7) - where MAP4K4 expression is high in certain cellular compartments also under physiological conditions.

### 1.1 MAP4K4 expression across tissues

**Figure 1:**
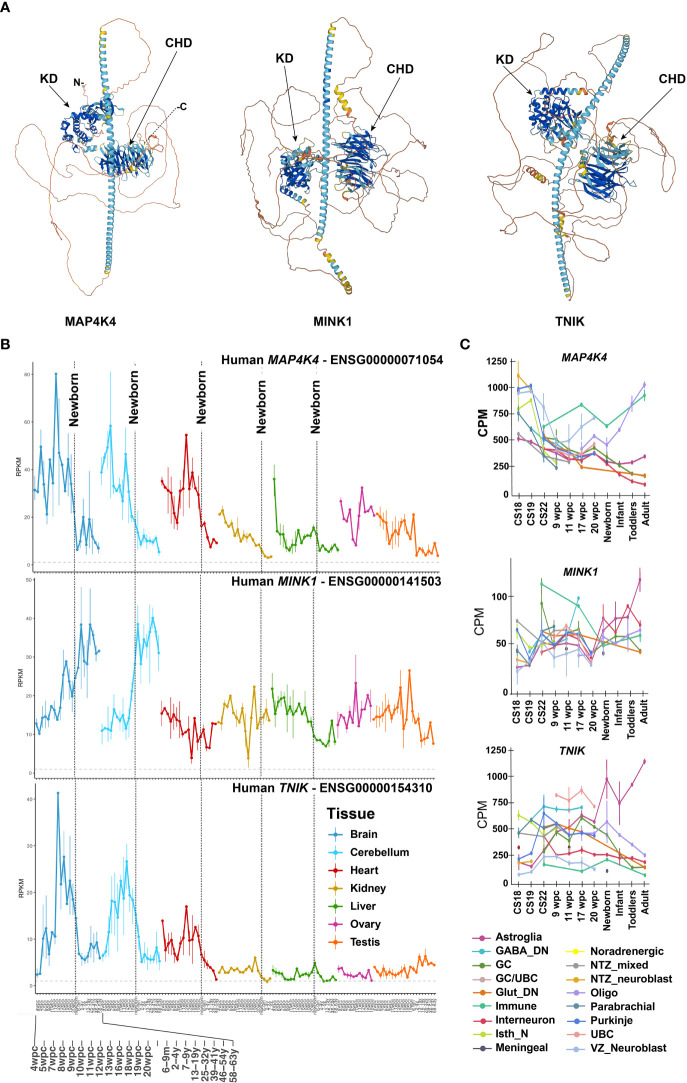
Protein structure and differential expression of *MAP4K4*, *MINK* and *TNIK* across tissues and developmental stages.**(A)** AlphaFold protein structures of *MAP4K4* (UniProt: E7ENQ1), *MINK1* (UniProt: Q8N4C8) and TNIK (UniProt: Q9UKE5) (11, 12). CHD, citron homology domain, KD, kinase domain. **(B)** *MAP4K4*, *MINK1* and *TNIK* single nucleus RNA sequencing expression profiles across organs and developmental stages (13). The X-axis depicts developmental stage before and age after birth. The vertical dotted lines indicate birth. The Y-axis are reads per kilobase of exon model per million mapped reads (RPKM). **(C)** Single nucleus mRNA expression analysis of *MAP4K4*, *MINK1* and *TNIK* across human developmental stages of the human cerebellum (14). CPM, Counts per million; GABA, Gamma-aminobutyric acid, DN, Dentate nucleus; GC, Granule cells; UBC, Unipolar brush cells; Glut_DN, Glutamatergic deep nuclei neurons; Isth_N, Isthmic nuclei neurons; NTZ, Nuclear transitory zone; VZ, Ventricular zone.

Despite the high potential of MAP4K4 as a disease-agnostic therapy target, identifying a therapeutic window for an efficacious, non-toxic therapy is challenging. High levels of *MAP4K4* RNA in brain, heart and testis were already detected by Northern Blot hybridization analysis some 25 years ago (1, 8, 9), indicating a particular physiological relevance of MAP4K4 in these tissues. Several isoforms resulting from splice variants of the *MAP4K4* gene located on chromosome 2, are differently expressed among tissues (1, 9), which may furthermore indicate variant-specific functions in the different locations (10). In total 15 alternative, protein-coding splice variants have been identified, but mechanisms of differential regulation or the functional significance of alternatively spliced MAP4K4 have not yet been addressed. The longest isoform is composed of 31 exons and measures 7458 base pairs, which are translated into 1320 amino acids. The two closest homologs of MAP4K4 are Misshapen-like kinase 1 (MINK1 or MAP4K6, 86.8% identical), located on chromosome 17, and TRAF2 and NCK-interacting kinase (TNIK or MAP4K7, 81.96% identical), located on chromosome 3 (**Figure 1A**). More recent analyses of RNA and protein expression (https://www.proteinatlas.org/) revealed a discrepancy between RNA and protein detection in the gastrointestinal tract, the liver, the bladder, in male tissues and in the brain. *MAP4K4* mRNA expression in the gastrointestinal tract, the liver, the bladder and in the male tissues is low and contrasts with higher expression at the protein level in these tissues. Conversely, in the brain, very high *MAP4K4* expression at mRNA level in oligodendrocytes, astrocytes and microglia contrasts only low protein expression, which is detected exclusively in the basal ganglia, the cerebral cortex and the cerebellum, indicating either the existence of splice variants that cannot be detected with the antibodies used or translational or post-translational control of *MAP4K4* in the brain. Gene expression analysis across organs (13) revealed high *MAP4K4* expression during developmental stages starting four weeks post conception (4 wpc, **Figure 1B**). During development, *MAP4K4* expression is highest in the cerebrum and the cerebellum, followed by the heart. At approximately 12 wpc, *MAP4K4* expression in the cerebrum and the cerebellum begins to drop sharply and reaches a plateau around 4 – 9 years of age. Interestingly, *MINK1* expression in the cerebrum and the cerebellum differs considerably from *MAP4K4*, as it gradually increases, starting from 4 wpc throughout embryonal development, early and middle life. *TNIK* expression peaks 12 wpc and declines similarly to MAP4K4 until 4 years of age, when it reaches a lower plateau. Single-cell nuclear pre-mRNA analysis during development revealed that *MAP4K4* mRNA expression is high across all neuronal cell types at Carnegie Stage (CS) 18, after which it declines to a minimal level at time of birth and later (14). Exceptions are the ventricular zone neuroblasts, where mRNA expression of *MAP4K4* increases between 9 and 22 wpc and oligodendrocytes, where *MAP4K4* expression continuously increases starting at 17 wpc (**Figure 1C**).

### 1.2 MAP4K4 function in non-cancerous tissues in health and disease

**Figure 2:**
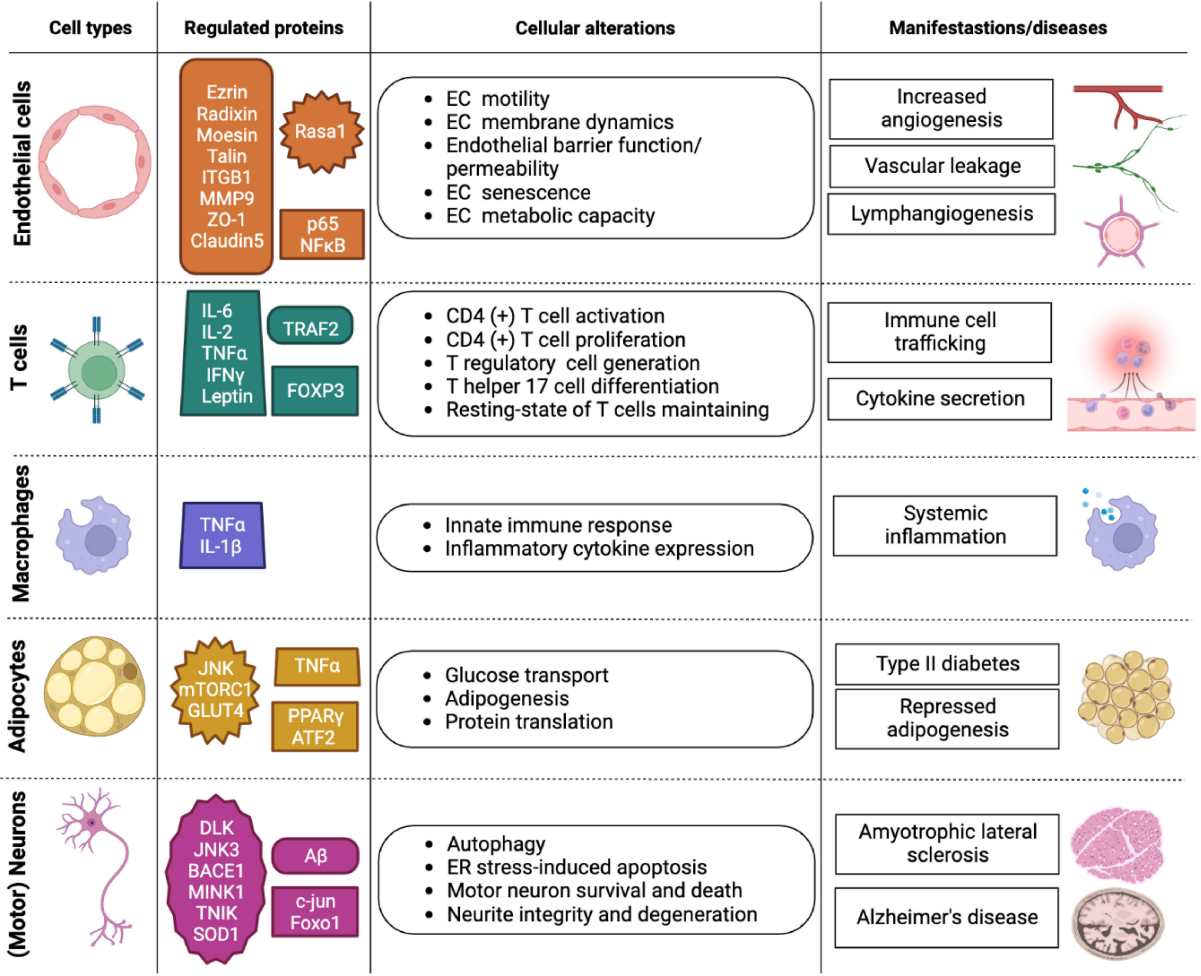
Overview of MAP4K4 functions in different cell types. Overview of MAP4K4 functions in endothelial cells, T cells, macrophages, adipocytes, and motor neurons. The column entitled regulated proteins highlights proteins that are either directly or indirectly regulated by MAP4K4 function. The column entitled cellular alterations states cellular activities affected by MAP4K4, and the column entitled manifestations/diseases indicates the corresponding physiological or pathophysiological contexts. EC, Endothelial cell; ER, Endoplasmic reticulum.

MAP4K4 is critically involved in regulating cell-autonomous tumor growth and cancer metastasis (see also chapters 3.3 and 3.4). Detailed reviews of MAP4K4 control of cytoskeleton regulation and cancer can be found in references (15) and (10), respectively. In addition, non-autonomous functions of MAP4K4 in the tumor stroma including the regulation of endothelial cells during vascular development, endothelial cell motility, T cell activation and of glucose up-take and lipogenesis may contribute to tumor growth and progression. In-depth analysis of MAP4K4 functions in physiological and non-cancer pathological processes could thus reveal molecular mechanisms of MAP4K4 control of cellular processes that – when de-regulated - could contribute to tumorigenesis. The following sections provide an overview of MAP4K4 functions in endothelial homeostasis, inflammatory signaling and glucose up-take, lipogenesis and neurological disorders (**Figure 2**). For additional, more in-depth information on MAP4K4 in inflammation and in metabolic and cardiovascular diseases, the reader is referred to references (16) and (17), respectively.

#### 1.2.1 MAP4K4 in endothelial cells

Carcinomas originate from endothelial tissues that have undergone oncogenic transformation and endothelial cells contribute to shaping the structural and functional landscape of the tumor microenvironment (18). MAP4K4 is an essential protein in endothelia and knockout (KO) of *Map4k4* is embryonically lethal in mice due to defects in mesodermal and endodermal cell migration (19). Hence, MAP4K4 contribution to overall tumor incidence in embryonal or adult tissue carcinomas can thus not be assessed. Conditional KO of *Map4k4* in endothelial cells of mice resulted in no surviving homozygous animals (20) and chylothorax (21), indicating an essential role of MAP4K4 in developing endothelia of blood and lymphatic vasculature. One function of MAP4K4 in endothelial cells is the control of membrane retraction by forcing the turnover of focal adhesions (FAs) (20). Mechanistically, MAP4K4 phosphorylation of the ERM protein moesin promotes its competitive binding to the C-terminus of β1 integrin (INTß1), causing talin displacement and impeded INTß1 activation, FA turnover and tail retraction, which are essential pre-requisites for the migration of endothelial cells. Consequently, mice with conditional KO of *Map4k4* in endothelial cells displayed impaired angiogenesis, which in the tumor context reduced tumor growth rate and vascular perfusion (20). During inflammatory tissue responses, MAP4K4 also promotes increased vascular permeability and leukocyte adhesion downstream of tumor necrosis factor alpha (TNFα) signaling (22), indicating a broader function of MAP4K4 in vascular biology that may contribute to pathogenesis and cancer. Consistently, MAP4K4 was also found to cause early blood-brain barrier damage in the subarachnoid hemorrhage model (23). In this model, increased MAP4K4 expression is associated with nuclear factor kappa B (NF- κB/p-p65) phosphorylation, matrix metalloproteinase 9 (MMP9) expression and the degradation of tight junction proteins zonula occludens 1(ZO-1) and claudin 5. These effects were reduced after the treatment with the MAP4K4 inhibitor PF-06260933 (23). Thus, non-autonomous functions of MAP4K4 in endothelial cells likely contribute to tumor progression by increased vascularization and an accentuated response to the inflammatory cytokine TNFα. Importantly, the approved BCR-ABL and MAP4K4 inhibitory drug bosutinib (Bosulif^®^) restores endothelial barrier functions and reduces vascular leakage (24), rendering this compound a candidate drug for tumor patients suffering from tumors associated with aberrant MAP4K4 functions.

#### 1.2.2 MAP4K4 in the immune cell compartment

Conditional KO of *Map4k4* in T cells does not affect the development of T cells, B cells, neutrophils and macrophages, but causes increased interleukin 6 (IL-6) and IL-17 expression and inflammation-associated disorders in different organs, with immune cell infiltration into skin, eyes and liver (25). T cell specific *Map4k4* KO in mice leads to the development of hepatic steatosis, and insulin resistance. This is in accordance with previous reports, which identified MAP4K4 as a negative regulator of adipocyte lipogenesis, adipogenesis and insulin-stimulated glucose transport (26, 27). Mechanistically, MAP4K4 phosphorylates TNF receptor-associated factor 2 (TRAF2) on Ser35, which leads to TRAF2 lysosomal degradation in T cells. Consequently, MAP4K4 restricts TRAF2 expression and thereby reduces IL-6 production (25). In the central nervous system (CNS), IL-6 can exert opposing actions and trigger either neuron survival after injury or cause neurodegeneration and cell death in neurodegenerative or neuropathic disorders (28). Furthermore, increased IL-6 expression is associated with numerous neuropathological changes through either direct signaling or trans signaling *via* proteolytically shed, soluble IL-6 receptor (29). MAP4K4 induces TNFα and IL-1 production in macrophages and concomitant systemic inflammation (30), thereby possibly triggering a positive feedback *via* TNFα-induced MAP4K4 expression (31, 32) and cell invasion in transformed cells (32–34). Finally, in CD4+ T cells KO for Map4k4, decreased IL-2 and interferon gamma (IFN γ) expression was noted (35). Hence, one function of MAP4K4 could involve the maintenance of immune cell balance, both by controlling cytokine expression (25, 30, 35) and immune cell trafficking across endothelial and lymphatic vessel walls (22). However, the systemic impact of the complex regulatory functions of MAP4K4 in different immune cell compartments and tissues remains incompletely understood, and the consequence of pharmacological repression of MAP4K4 functions, as envisaged for anti-cancer therapies, is complex and needs to be carefully assessed.

#### 1.2.3 MAP4K4 in adipocytes

MAP4K4 was identified in adipocytes as a negative regulator of insulin-responsive glucose transport downstream of TNFα through reduction of the expression of the glucose transporter isoform 4 (GLUT4) (27). On the one hand, MAP4K4 thereby suppresses GLUT4 and the adipogenic transcription factors CCAAT/enhancer-binding protein alpha or beta (C/EBPα, C/EBPβ) and peroxisome proliferator-activated receptor gamma (PPARγ) (27). On the other hand, MAP4K4 promotes excessive JNK, extracellular regulated kinase (ERK-1/2) and insulin receptor substrate 1 (IRS-1) phosphorylation (27). Depletion of MAP4K4 early in adipocyte differentiation enhances adipogenesis and triglyceride deposition, and in fully differentiated adipocytes, MAP4K4 loss up-regulates GLUT4 (27). MAP4K4 inhibits mTORC1 activity *via* AMP-protein kinase (AMPK) signaling and the phosphorylation of mTORC1 binding partner raptor (36). As a consequence, the phosphorylation of 4E-BP1 is reduced and leads to repressed translation of *PPARγ* (36). The resulting reduced expression of PPARγ, combined with the suppression of sterol regulatory element-binding transcription factor 1 (SREBP-1), contributes to the MAP4K4-dependent repression of lipogenesis (26). The current knowledge thus indicates that MAP4K4 control of lipogenesis and glucose uptake (37) is most likely associated with the transcriptional repression of proteins associated with glucose uptake and lipogenesis, and not through direct control of these proteins at posttranscriptional level.

#### 1.2.4 MAP4K4 in the nervous system and neurodegenerative diseases

MAP4K4 displays relatively higher expression levels in the brain (9, 38). Further up-regulation of MAP4K4 compared to corresponding normal tissue controls has been noted in the aggressive primary brain tumors glioblastoma and medulloblastoma (1, 6, 39). The predominant expression of MAP4K4 in neuronal tissues raises the question as to whether MAP4K4 in primary brain tumors is implicated in similar or related pathophysiological processes as in neurodegenerative disorders, and whether we could learn from MAP4K4 functions in neurodegeneration to better treat brain tumor patients in the future. Indeed, cancer and neurodegenerative diseases display common pathophysiological alterations such as aberrant cell cycle control, redox imbalance, tissue inflammation and immune responses, although the net outcome for the former is an increased replicative potential combined with an efficient protection from apoptotic stimuli, whereas upregulation of cell cycle regulators in latter results in progressive degeneration and cell death (40, 41).

Single-cell mRNA expression analysis of pre-frontal cortex tissue of Alzheimer’s disease (AD) patients identified increased MAP4K4 expression as a potential biomarker of the disease (42). In AD, upstream β-secretase 1 (BACE1) expression and amyloid-β (Aβ) deposition are driven by ectopic oxidative stress *via* multiple mechanisms, including c-Jun N-terminal kinase (JNK) activation (43, 44). It was thus postulated that oxidatively damaged DNA might induce MAP4K4, thereby sensitizing the brain to oxidative stress-induced JNK activation and BACE1 induction (45).

Unlike AD, where no functional studies using agents targeting MAP4K4 have been performed so far, MAP4K4 inhibition by the small molecule dual MAP4K4-GSK3 inhibitor kenpaullone was found to be motor-neuron protective in amyotrophic lateral sclerosis (ALS) (46). Consistent with a pro-apoptotic function of MAP4K4 in ALS, specific inhibition of MAP4K4 with experimental Compound 29 (47) prevented motor neuron death and improved neurite integrity both by directly blocking JNK3-cJUN-mediated apoptosis and by activating FOXO1-mediated autophagy (48). Neuronal stress induced by withdrawal of trophic factors triggers pro-apoptotic functions of germinal center kinases (GCK) IV MAP4K4, MINK1 and TNIK in embryonic dorsal root ganglion neurons (49). Depletion of these three kinases simultaneously or treatment of the stressed neurons with the second generation MAP4K4 inhibitor GNE-495 protected the neurons from apoptosis induced by dual leucine zipper kinase (DLK) activation and JNK-dependent phosphorylation of c-Jun (49). The notion that MAP4K4 may be a new target for the treatment of neurodegenerative diseases was already previously postulated in a study using optimized small molecule compounds based on the neuritogenic pyridones (militarinone-inspired 4-hydroxy-2-pyridone) collection (50). The most potent 4-hydroxy-2-pyridone identified in this study is a selective ATP-competitive inhibitor of MAP4K4 that does not repress other stress pathway-related kinases. More recently, a screen for compounds protecting motor neurons from endoplasmic reticulum (ER) stress-induced apoptosis found that compounds targeting MAP4K4 were most effective. Lead structure-based compound design combined with functional testing identified the Prostetin/12k MAP4K4 inhibitor as an exceptionally potent neuroprotective molecule (46). Due to the essential role of MAP4K4 during neuro-pathophysiological processes, this molecule could be of particular relevance for targeting aberrant MAP4K4 functions in brain tumors as its metabolic stability and blood-brain barrier permeance was confirmed. This and other small molecule MAP4K4 inhibitors are listed in **Table 1**. Despite the high potential clinical relevance of selective MAP4K4 inhibition in invasive cancers, only for Prostetin 12/K is currently a clinical trial open to assess safety and pharmacokinetics of this drug.

**Table 1:** Inhibitors of MAP4K4 used in different disease studies.

Name	Target	Effects in Disease/Syndrome	Ref
*GNE-220*	MAP4K4 MAP4K5 (KHS1), MAP4K6 (MINK1), DMPK	Regulation of protrusion formation in human umbilical vein endothelial cells (HUVECs). Suppression of pathological angiogenesis in mice.	(20)
*GNE-495*	MAP4K4	Inhibition of migration and invasion in neuron protection medulloblastoma. Suppression of retinal angiogenesis.	(6) (20) (49) (51)
*PF-06260933*	MAP4K4	Reduction in glucose levels, amelioration of plaque development and/or plaque reduction *in vivo.* Reduction of blood-brain barrier damage in mice, improved neurological recovery.	(23)
*4-Hydroxy-2-pyridone*	MAP4K4	Enhancement of neurite outgrowth.	(52)
*6-(2-fluoropyridin-4-yl)pyrido[3,2-d]pyrimidin-4-amine, Compound 29*	MAP4K4	Improved survival of motor neurons. Repression of stress-induced retrograde JNK signaling and protection from neurodegeneration. Pharmacodynamic effects in a human tumor xenograft model.	(47–49)
*Kenpaullone*	CDK1/cyclin B, GSK-3β, MAP4K4, KLF4, CDK2/cyclin A, CDK2/cyclin E, and CDK5/p25	Inhibition of tumor cell growth. Maintenance of pancreatic β cell survival. Improved cardiomyocyte survival.	(53, 54)
*Prostetin/12k*	MAP4K4	Protection of motor neurons from ER stress-induced apoptosis in amyotrophic lateral sclerosis (ALS).	(46)
*Bosutinib (Bosulif)*	BCR-ABL, MAP4K4	Protection against inflammation-induced endothelial barrier disruption. Prevention of vascular leakage and attenuation of murine alveolar protein leakage and pulmonary edema.	(24)
*DMX-5804*	MAP4K4 MAP4K6 (MINK1), MAP4K7 (TNIK)	Prevention of oxidative-stress in cardiac myocyte cell death. Reduction of ischemia-reperfusion injury in mice.	(54)

Further in-depth investigations of the divergent molecular functions of MAP4K4 activity during onset and progression of brain tumors and neurodegeneration will be necessary to design disease-specific intervention strategies targeting differential MAP4K4 functions in these two disease entities.

### 1.3 MAP4K4 control of migration and invasion

MAP4K4 was identified as a broadly overexpressed kinase in human tumor cells (1) and has since then been recognized as an emerging target in cancer (10), in particular in solid tumors of epithelial tissues. Loss of function studies in gastric (55), hepatocellular carcinoma (56), prostate cancer (34) and pancreatic cancer cells (57) indicated tumorigenic and growth-promoting activities of MAP4K4. However, some of the described effects might be an indirect consequence of kinase function or – in the cases where miRNAs controlling MAP4K4 were investigated – of additional targets affected by the same miRNA. Hence, after the identification of MAP4K4 as a promigratory kinase in tumor cells (58), the focus of the field shifted to the role of MAP4K4 in the control of cytoskeleton dynamics and cell motility. The mouse ortholog of MAP4K4 – NCK-interacting kinase NIK – controls actin dynamics and cell motility through direct interaction and regulation of the actin-related protein 2 (ARP2) (59) and of proteins of the ezrin, radixin, moesin (ERM) family (60). The combined activity of MAP4K4 and ERM proteins towards actin dynamics and cytoskeleton reorganization, respectively, increases invasiveness downstream of TNFα by promoting the formation of membrane protrusions at the leading edge (32, 33). Phosphorylation of moesin by MAP4K4 in endothelial cells also enables membrane retraction in the trailing edge and thus migration of these cells by competing for talin binding to the β1-integrin intracellular domain and by prompting focal adhesion (FA) destabilization (20). MAP4K4 also promotes FA destabilization in skin cells by microtubule-guided activation of ARF6 *via* the guanine nucleotide exchange factor IQ motif and SEC7 domain-containing protein 1 (IQSEC1) (61). Conversely, MAP4K4 also promotes the activation of integrin β1 at the leading edge of invading cells stimulated by hepatocyte growth factor (HGF) (6), suggesting differences in regulation and effector interactions of MAP4K4 in leading and trailing edge cell compartments. In adult patient-derived glioma cells, MAP4K4 is necessary for pro-metastatic functions (7), and in pediatric medulloblastoma cells, it promotes invasion induced by epidermal (EGF), hepatocyte (HGF) and basic fibroblast growth factor (bFGF) (6, 39, 62). In addition to the direct regulation of cytoskeleton modulatory proteins such as ARP2, ERM proteins and VASP, MAP4K4 also influences biological processes that indirectly control migratory and invasive behaviors of cells. Examples for latter are the increase in intracellular pH *via* the sodium proton exchanger NHE1 (63) or the regulation of endocytosis (64). In this context, MAP4K4 was found necessary for maintaining the surface expression of the poliovirus receptor/CD155 (PVR/CD155) after growth factor stimulation in medulloblastoma, as depletion of MAP4K4 resulted in reduced plasma membrane association of PVR/CD155 in HGF-stimulated cells derived from this tumor. Importantly, the depletion of PVR/CD155 reduced the speed of migration, proliferation and tissue invasion of the tumor cells (64). Thus, although mechanistically incompletely understood, these observations highlight the implication of MAP4K4 in processes controlling the abundance, availability and activity of cell surface proteins, which considerably increases reach and repertoire of cell fate modulation by this kinase.

In conclusion, MAP4K4 is a well-established regulator of cytoskeleton dynamics, cell motility and invasiveness, and this function of MAP4K4 is mediated by direct and indirect effector mechanisms controlled by phosphorylation, protein-protein interactions and the spatial regulation of protein activities.

### 1.4 Cooperation of STRIPAK and MAP4K4 to control pro-invasive and anti-proliferative activities in tumor cells

#### 1.4.1 Proliferation control through Hippo pathway activation

The identification of GCKs III and IV as components of the multiprotein striatin-interacting phosphatase and kinase (STRIPAK) complex (65) began to shed light on a novel function of MAP4K4 and related GCKs, and it led to the discovery of the antiproliferative activity of MAP4K4 *via* the activation of Hippo tumor suppressor signaling (3, 66, 67). The activation of the Hippo tumor suppressor pathway results in the phosphorylation, cytosolic retention and proteasomal degradation of the transcriptional co-activators YES-associated protein YAP65 homolog (YAP) and transcriptional co-activator with PDZ-binding motif (TAZ). In the non-phosphorylated state, YAP and TAZ enter the nucleus and coactivate the transcriptional enhanced associate domain (TEAD1/2) transcription factors (68). MAP4K4 and the related MST1/2 kinases phosphorylate the hydrophobic pocket of the large tumor suppressor kinases 1 and 2 (LATS1/2), which in turn phosphorylate YAP and TAZ (3), leading to their cytoplasmatic retention and proteasomal degradation (68). Thus, activated MAP4K4 causes reduced nuclear accumulation of YAP and TAZ, and concomitantly reduced expression of TEAD1/2 target genes, which are involved in in cell fate determination, cell polarity, proliferation, and survival. One layer of control of MAP4K4 activity towards LATS1/2 is the dephosphorylation of MAP4K4 by STRIPAK-associated protein phosphatase 2A (PP2A), for which striatins (STRN3 and STRN4) act as regulatory subunits (69). STRN3/4 thereby recruits the STRIPAK-associated kinases MST1 and MST2 or MAP4K4 and enables their dephosphorylation by PP2A (66, 69). The concomitant inactivation of the Hippo pathway results in reduced phosphorylation of YAP and TAZ, and increased TEAD1/2 target gene expression. The concurrent recruitment of MAP4K4 and PP2A on STRN3/4 is prevented when neuro-fibromatosis 2 (NF2)/merlin and kidney and brain protein (KIBRA) are bound to striatins in serum-starved cells (70). Cellular stimulations leading to the activation of the small GTPase Rho cause the displacement of NF2/merlin and KIBRA from striatins and to the recruitment of PP2A through the STRIPAK member STRIP1 (67). In consequence, MST1/2 kinases or MAP4K4 are dephosphorylated, and the Hippo pathway is switched off. YAP and TAZ activation is also regulated independently of growth factor abundance by matrix stiffness through the distribution and abundance of FAs and cell contractility (71, 72). Low stiffness leads to the activation of the Hippo pathway resulting in the phosphorylation of LATS1/2, YAP and TAZ (73). This low stiffness-induced Hippo pathway activation is mediated by the small GTPase RAP2, which directly interacts with MAP4K4 *via* its citron homology domain (CNH) (74), and thereby acts as a mechanotransducer between integrin-activated phospho-lipase Cγ (PLCγ) and the Rho GTPase activating protein 29 (ARHGAP29) (73). In conclusion, low serum or low stiffness lead to decreased association of MAP4K4 with the dephosphorylating activity of PP2A on the STRIPAK complex, which results in Hippo pathway activation and reduced cell growth. Conversely, increased serum or increased matrix stiffness with concomitant activation of Rho triggers the dephosphorylation of MAP4K4 by PP2A, which stalls Hippo pathway activation and contributes to cell growth and survival. In these processes, the STRIPAK complex takes center stage, as it enables and coordinates the interaction of PP2A with MAP4K4.

#### 1.4.2 Invasion control through PKCθ and VASP

How can the tumor-suppressive activity of MAP4K4 *via* Hippo pathway activation be reconciled with the tumor-promoting activity of MAP4K4 through increasing actin and cytoskeleton dynamics? Does the STRIPAK complex also control the invasion-promoting activities of MAP4K4? Although the mechanistic details of STRIPAK control of MAP4K4 invasion-promoting activities are still not fully elucidated, STRN3 and MAP4K4 were recently found to co-operate towards increased invasiveness induced by the growth factor bFGF (62). Specifically, bFGF-induced invasion was found to depend on MAP4K4-mediated phosphorylation and activation of PKCθ, and in part on the actin modulator vasodilator-stimulated phosphoprotein (VASP) (62). The activation of both PKCθ and VASP by MAP4K4 depends on its association with the STRIPAK complex *via* the CNH domain of MAP4K4, which highlights the relevance of multiprotein complex regulation of MAP4K4 function. Both activities are independent of PP2A, suggesting that the function of PP2A towards MAP4K4 is restricted to Hippo signaling. Interestingly, MAP4K4-dependent phosphorylation of PKCθ was also noted in CD4+ T cells stimulated with phorbol ester/Ionomycin (35), suggesting that PKCθ is a general downstream effector kinase of MAP4K4.

The STRIPAK component STRIP1 is dispensable for coupling STRN3/4 and MAP4K4 (62, 67). However, depletion of STRIP1 increases the MAP4K4 activation signature and the invasive potential of tumor cells (62), suggesting a negative regulatory role of STRIP1, possibly by making MAP4K4 accessible to PP2A. Striatins are highly expressed in the cerebrum and the cerebellum. In the cerebellum, striatins are specifically detected in neuronal cell bodies and dendritic spines but not in axons (75), indicating a key role in neuronal development or neuronal physiology. High expression of STRN3 and STRN4 in the cerebellar tumor medulloblastoma correlates with increased phosphorylation of S540 and S608 in the interdomain of MAP4K4 and decreased phosphorylation of C-terminal S873 and S778 (76). PP2A activity is directed against Ser and Thr residues in the C-terminus of MAP4K4 (66). Thus, the phosphorylation of these residues as a consequence of MAP4K4 dissociation from PP2A may be necessary for MAP4K4 activity towards Hippo pathway activation, but not for enabling the promigratory phosphorylation of PKCθ, which is independent of PP2A (62). Further studies will be necessary to disentangle the spatio-temporal regulation of STRIPAK-associated MAP4K4 activity towards invasion control and the precise role of the various STRIPAK complex components in this process.

### 1.5 Upstream regulators and downstream effectors of MAP4K4

**Figure 3:**
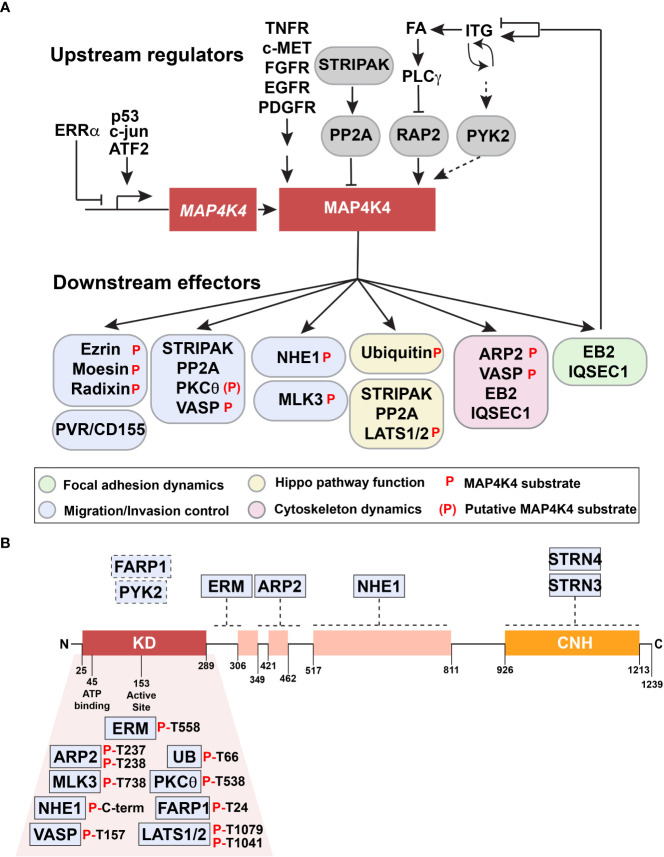
Upstream regulators and downstream effectors of MAP4K4. **(A)** Upstream regulators and downstream effectors of MAP4K4 are shown. Solid arrows or capped lines indicate experimentally established regulation, dotted arrows indicate potential regulatory mechanism. **(B)** Proteins indicated in boxes above the structure of MAP4K4 are experimentally confirmed interactors of MAP4K4. Dotted lines indicate approximate binding regions on MAP4K4. Proteins indicated below the kinase domain are experimentally validated substrates of MAP4K4. Residues phosphorylated by MAP4K4 are indicated.

Toxicities associated with the blood-brain barrier penetration of pyridopyrimidine MAP4K4 inhibitor 1 upon repeated dosage (51) raised concerns about general toxicities associated with MAP4K4 inhibition in the CNS. More recent discoveries of neuroprotective activities of neuritogenic pyridones (50) and of the MAP4K4 inhibitor Prostetin/12K (46) partially alleviated these concerns. However, indirect strategies to repress MAP4K4 effector functions could further contribute to an improved and more specific control of MAP4K4-dependent disorders. The following chapter summarizes proteins for which interaction with MAP4K4 was experimentally validated (**Table 2**, **Figure 3A**). For clarity, these proteins are grouped in upstream regulators (3.5.1.) and downstream effectors (3.5.2.) of MAP4K4.

**Table 2:** Summary of interactors and substrates of MAP4K4.

Interactor	Function	References
ARP2	NIK (murine MAP4K4) binds and directly phosphorylates the ARP2 subunit, which enables and increases the nucleating activity of the ARP2/3 complex.	(59)
EB2	MAP4K4 regulates focal adhesion turnover and cell motility by binding to EB2, a microtubule-binding protein, and by binding to IQSEC1, a guanine nucleotide exchange factor to enhance focal adhesion turnover.	(61)
Ezrin	MAP4K4/NIK phosphorylates ezrin on the C-terminal regulatory Thr residue and promotes cell motility and invasiveness.	(32, 60)
FARP1	MAP4K4 binds and phosphorylates FARP1. FARP1 contributes to neurite formation and is necessary for dendrite branching.	(77)
IQSEC1	MAP4K4 regulates focal adhesion turnover and cell motility by binding to EB2, a microtubule end binding protein, and by binding to IQSEC1, a guanine nucleotide exchange factor to enhance focal adhesion turnover.	(61)
LATS1/2	MAP4K4 phosphorylates LATS1/2, resulting in YAP/TAZ phosphorylation, cytoplasmatic retention and proteasomal degradation. This function constitutes a core mechanism of Hippo tumor suppressor pathway activation.	(3)
MLK3	MAP4K4 and MLK3 interact and MAP4K4 phosphorylates MLK3 on Thr738, which increases MLK3 kinase activity and downstream signaling. Phosphorylation of MLK3 by MAP4K4 promotes pancreatic cancer cell proliferation, migration, and colony formation.	(78)
PYK2	MAP4K4 as a binding partner of PYK2, which phosphorylates MAP4K4 on Tyr residues. The cooperation of MAP4K4 and PYK2 promotes glioma cell migration.	(79)
PKCθ	The cooperation of MAP4K4 and STRN3 in the STRIPAK complex promotes a pro-migratory and invasive phenotype in medulloblastoma cells through the activation of downstream effector PKCθ.	(62)
PP2A	MAP4K4 interacts with PP2A in the STRIPAK complex to control cytoskeleton dynamics in cardiomyocytes and Hippo pathway activation in various cell types.	(62, 66, 80)
RAP2	RAP2 GTPase is activated by low extracellular matrix stiffness. Activated RAP2 binds to MAP4K4/6/7, resulting in Hippo pathway activation and MAP4K4/6/7-dependent ubiquitin phosphorylation.	(73, 74)
Radixin	MAP4K4 phosphorylates radixin and promotes cell motility and invasiveness.	(60)
STRN3 STRN4	MAP4K4 binds STRN4, a component of the STRIPAK complex. STRIPAK signaling activates the Hippo pathway *via* MAP4K4 on the one hand and promotes pro-invasive signaling *via* PKCθ on the other hand. Dysregulated STRIPK signaling is involved in cancer progression due to impaired Hippo tumor suppressor pathway activation and increased invasiveness.	(62, 66)
STRIP1	MAP4K4 interacts with STRIP1, a component of the STRIPAK complex. STRIP1 is involved in MAP4K4 repression *via* PP2A.	(62, 66)
TRAF2	MAP4K4 downregulates IL-6 production in T cells through direct phosphorylation and degradation of TNF receptor-associated factor 2 (TRAF2), leading to the suppression of Th17 cell-mediated insulin resistance *in vivo*.	(25)
Ubiquitin	MAP4K4/6/7 kinases phosphorylate ubiquitin in cells at low extracellular matrix stiffness, leading to decreased DNA repair and increased sensitivity to DNA-damaging agents.	(81)
VASP	MAP4K4 phosphorylates VASP_S157_. S157 phosphorylation depends on MAP4K4 interaction with STRN3 *via* the CNH domain and is associated with cell invasiveness.	(62)

Table summarizing activators and effectors of MAP4K4 and the biological function of their interaction with MAP4K4.

#### 1.5.1 Upstream regulators of MAP4K4

Receptor tyrosine kinase (RTK) activation can act as an upstream event of MAP4K4 induction and activation, through transcriptional upregulation *via* c-Jun and activating transcription factor 2 (ATF2) as well as *via* transcription-independent activation control. However, the exact mechanism of MAP4K4 activation such as for example allosteric regulation of the conformation, phosphorylation of activating residues or other posttranscriptional processes remains still unclear (31, 32, 39, 60, 64). In contrast, MAP4K4 repression through dephosphorylation by PP2A has been confirmed in several independent studies (62, 66, 67, 82). Downstream of FA, MAP4K4 activity is induced through the small GTPase RAP2, which interacts with MAP4K4 (74) and TNIK (83) *via* their respective CNH domains. Under conditions of low matrix stiffness, RAP2 is activated through GDP-GTP exchange and promotes an electrophoretic mobility shift in MAP4K4, which indicates MAP4K4 activation through phosphorylation. Activated MAP4K4 then phosphorylates the Hippo kinase LATS1 and its downstream target YAP (73), which leads to its cytoplasmatic retention and proteolytic degradation. This constitutes a mechanism of mechanical regulation of Hippo pathway activation and repression of YAP/TAZ target gene expression, complementing the previously reported confinement of YAP/TAZ target gene expression to cells experiencing mechanical stresses (71). Mechanistically, RAP2 activation depends on the conversion of phosphatidyl-inositol 4,5 bi-phosphate (PIP2) to phosphatidic acid (PA) by phospholipase D1/2 (PLD1/2) and subsequent PA-dependent postsynaptic density, disc-large, zonula occludens GTP exchange factor 1,2 (PDZGEF1,2) mediated GTP loading of RAP2 (73). PA production from Phosphatidylinositol 4,5 (PI4,5) and downstream GTP-loading of RAP2 is repressed by phospho lipase Cγ (PLCγ). PLCγ is activated through focal adhesions, which provides a mechanistic link between adhesion and Hippo pathway activation. Thus, FA induction and the concomitant generation of mechanical stress triggers RAP2 activation, followed by MAP4K4-mediated phosphorylation of LATS1,2, hippo pathway activation and growth repression. This process is stalled by PLCγ activation, which reduces PA production and subsequent Hippo pathway activation through RAP2-MAP4K4 signaling. PLCγ activation is also induced by growth factor signaling, suggesting that targeted inhibition of PLCγ could result in growth repression through Hippo pathway activation. Novel allosteric inhibitors with improved specificity and potency against PLCγ are being developed (84), which may be explored in the future to drive RAP2-MAP4K4 mediated Hippo pathway activation and growth repression in growth factor activated tumors.

A yeast two-hybrid screen for the identification of interactors of the 4.1, ezrin, radixin, moesin (FERM) domain of proline-rich tyrosine kinase 2 (PYK2) identified MAP4K4 as a binding partner (85). Using a heterologous expression system, the same study revealed that PYK2 could tyrosine phosphorylate MAP4K4 and cooperatively increase migration of glioma cells. Although the MAP4K4-PYK2 interaction was not confirmed in subsequent proteomic analyses, this study indicates that tyrosine phosphorylation of MAP4K4 could constitute a regulatory mechanism to control its activity.

In a search for mediators of estrogen-related receptor alpha (ERRα) controlled actin cytoskeleton dynamics, *MAP4K4* was identified as an ERRα-repressed gene (86). Depletion of ERRα in the breast cancer line MDA-MB231 cause MAP4K4 up-regulation, increased phosphorylation of the ERM protein moesin and aberrant FA formation with concomitantly reduced adhesion to collagen I. Consistent with the regulatory function of MAP4K4 in integrin activation and adhesion (6, 20), pharmacological inhibition of MAP4K4 with PF-06260933 rescued adhesion in ERRα-depleted MDA-MB231 cells. MAP4K4 in these cells also contributes to prolonged ERM protein phosphorylation downstream of TNFα, and to increased TNFα-induced matrigel invasion (33). ERRα is thus the first transcriptional control element acting as a repressor of *MAP4K4* expression, unlike c-Jun and ATF2 (31), p53 (87) or SOX6 (88), which were identified as activators of *MAP4K4* transcription.

#### 1.5.2 Downstream effectors of MAP4K4

Several interactors and effector proteins downstream of MAP4K4 have been identified so far (**Figures 3A, B**), most of them are associated with the control of cell adhesion and cell migration. The first protein identified as MAP4K4 substrate was the sodium proton exchanger NEH1, to which the murine homolog of human MAP4K4, Nck-interacting kinase (NIK), was found to bind (63). NIK binding to the calcineurin homologous protein (CHP) domain of NHE1 requires amino acids 512 – 820 in the interdomain of NIK and is necessary for C-terminal NHE1 phosphorylation by NIK. C-terminal NHE-1 phosphorylation is induced downstream of growth factor stimulation, causing increased NHE1 activity and concomitantly increased intracellular pH (63). Thus, MAP4K4 couples growth factor activation to local pH regulation, which affects cell migration control through the activation of the GTPase Cdc42 (89) and the actin severing and caping protein cofilin (90).

Shortly after, the ERM family members ezrin and moesin were identified as interactors and substrates of MAP4K4, whereby the MAP4K4-mediated phosphorylation of the C-terminal regulating threonine residue T558 (for moesin) was found to be necessary for growth factor-induced lamellipodium formation (60). ERM proteins interact with MAP4K4 *via* the FERM domain, and a sequence N-terminal of the NHE1 binding site and corresponding to the region between amino acids 288 and 321 of the murine MAP4K4 homologue NIK is sufficient for ERM binding (60) (**Figure 3B**). Direct binding of ERM proteins to NHE1 was previously noted and the interaction of ERM proteins with NHE1 associated with the regulation of the cortical cytoskeleton. NHE1 was thereby suggested to act as an anchor localized in the plasma membrane and ERM proteins as its linker to the actin cytoskeleton (91). Thus, the complex constituted of NHE1, ERM proteins and MAP4K4 could act as a membrane-anchored signaling complex that promotes signal transmission and cortically anchored, localized actin polymerization (92). In endothelial cells, the phosphorylation of moesin by MAP4K4 also competes with talin for binding to α5β1 integrins and causes integrin inactivation, which is necessary for FA turnover and cell motility (20). MAP4K4 can also control FA turnover in an ERM-independent manner when recruited to microtubule tips through the interaction with the end binding protein 2 (EB2). MAP4K4 is targeted to FA through the EB2 complex, where it interacts with IQSEC to activate ARF6 (61). This control of FA turnover by MAP4K4 depends on intact microtubules, and the repression of microtubule polymerization with nocodazole halts FA disassembly. This is consistent with previous studies describing the necessity of microtubules for delivering essential proteins to the cell cortex (61, 93).

Another substrate of MAP4K4 in the context of migration is the actin-related protein 2 (ARP2), and MAP4K4 directly binds and phosphorylates ARP2 in response to growth factor stimulation (59). Residues 350-500 of MAP4K4 are sufficient for ARP2 binding and alanine substitution of T237 and T238 impair MAP4K4-mediated ARP2 phosphorylation. ARP2/3 phosphorylation was found to lock the complex in an active state. Consequently, dephosphorylation by phosphatase treatment inhibits its actin polymerization activity (94). Importantly, ARP2/3 complex phosphorylation by NIK restores actin polymerization activity of experimentally dephosphorylated ARP2/3. This suggests that the phosphorylation of the ARP2/3 complex by NIK is a means of controlling actin filament polymerization downstream of growth factor receptor signaling.

The STRIPAK complex contains striatins (STRNs) that act as regulatory subunits for PP2A to modulate kinase signaling cascades (65). Dysregulated STRIPAK signaling has been observed in cancer (95). Several components of the STRIPAK complex have been identified to directly interact with MAP4K4 (62, 66, 80). In cardiomyocytes, MAP4K4 interacts with PP2A and STRN/STRN3/STRN4 (80), and in medulloblastoma, with STRN3 and STRN4 (62). In medulloblastoma, the MAP4K4-STRIPAK complex interaction promotes growth factor-induced phosphorylation of PKCθ on T538 and this activation of PKCθ is necessary for tumor cell invasion. PKCθ activation depends on membrane localization and conformational changes induced and mediated by diacylglycerol (DAG) and receptor for activated kinases (RACK) (96). RACK is a member of the tryptophan-aspartate repeat (WD-repeat) family of proteins and it is involved in subcellular localization of proteins (97), and it was identified as a weak interactor of MAP4K4 (62). This suggests that RACK could bring MAP4K4 into proximity of PKCθ to phosphorylate and activate PKCθ in the context of RTK-activated PLCγ and increased DAG production. The cooperation of the MAP4K4-STRIPAK complex is also necessary for the direct phosphorylation of VASP on T157 by MAP4K4 (62). VASP is involved in F-actin filament elongation, FA maturation and cell motility, but the significance of VASP T157 phosphorylation besides membrane translocation for migration control is not fully understood.

In pancreatic cancer cells, MAP4K4 interacts with mixed lineage kinase 3 (MLK3) and thereby phosphorylates it on Thr738 (78). Phosphorylation of MLK3 increases its activity and promotes tumor cell proliferation and migration. Treatment with MAP4K4 inhibitor GNE-495 reduced these tumor-promoting activities associated with MLK3 phosphorylation *in vitro*, and reduced tumor weight and MAP4K4 and MLK3 expression *in vivo* (78).

A recent study found that low matrix stiffness increases the sensitivity to DNA-damaging drugs, and identified MAP4K4/6/7-dependent phosphorylation of ubiquitin on T66 as the underlying mechanism (81). This suggests that RAP2 activated by low matrix stiffness not only promotes Hippo pathway signaling and proliferation arrest through MAP4K4 (73), but also prevents the efficient repair of DNA breaks (81). It was thus suggested to pharmacologically activate MAP4K4 in combination with chemotherapeutic drugs to boost the cytotoxic response in the tumor cells.

In an effort to better characterize the substrate landscape of MAP4K4, the phosphoproteome of the liver cell line HepG2 treated with the germinal center kinase and MAP4K4 inhibitor Compound **1** was determined and compared to a list of proteins with increased phosphorylation after exposure to the kinase domain of MAP4K4 (77). The top candidate of this combined analysis is FERM, ARHGEF and pleckstrin domain-containing protein 1 (FARP1), which is phosphorylated by MAP4K4 on T24, thereby possibly contributing to neurite outgrowth and branching. This protein was also identified as a weak interactor of MAP4K4 (62). Despite the wealth of informative data generated in this study, it also highlights the difficulty of defining “true” and “unique” substrates of kinases associated with signaling complexes. And it was noted that one consequence of using the kinase domain of MAP4K4 to identify substrates was that the assay probed the intrinsic, active site-based specificity of MAP4K4 rather than the specificity created by protein−protein interactions or the structures of multiprotein complexes.

In conclusion, the activation of the effectors by MAP4K4 results in a paradoxical cell state, where on the one hand, growth is repressed and the cells are sensitized for apoptosis, and on the other hand, the migratory and invasive capability of the cells is increased. This obviously incites the challenging questions of whether and how modulation of MAP4K4 could be turned into anticancer therapies, and whether such therapies would need to be specifically tailored to the tissue of origin of the tumor.

### 1.6 Novel interactors of MAP4K4

**Figure 4:**
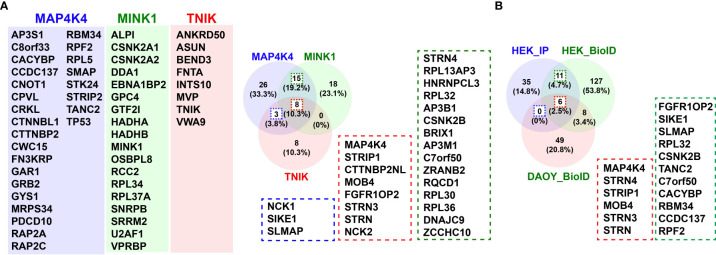
Interactors of MAP4K4. **(A)** Venn diagram of comparison of interactors of MAP4K4, MINK1 and TNIK identified by tandem affinity purification (5). 26 of the 52 proteins identified in the MAP4K4 pull-down were unique to MAP4K4, 15 also found in the MINK1 pull-down (green dotted line) and 3 found in the TNIK pull-down (blue dotted line). 8 of the proteins identified in the MAP4K4 pull-down were found both in the MINK1 and the TNIK pull-down (red dotted line). **(B)** Venn diagram of comparison of MAP4K4-associated interactors of MAP4K4, identified by tandem affinity purification (5) and proteins identified by proximity ligation (62). 11 (17%) of the proteins were detected in HEK293T cells both by pull-down and proximity ligation (green and red dotted lines). 6 proteins were detected both in HEK293T and DAOY cells (red dotted line), 8 proteins were detected by proximity ligation in both HEK293T and DAOY but not by tandem affinity purification.

**Figure 5:**
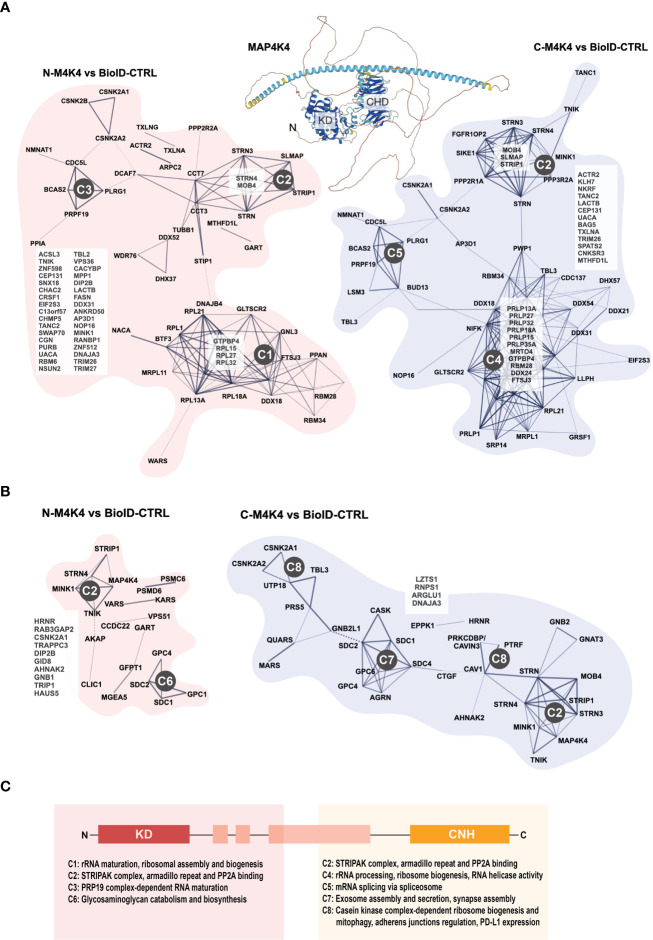
Protein interaction clusters of MAP4K4. **(A)** STRING protein interaction database analysis of proteins identified as MAP4K4 interactors in HEK293T cells using N- or C-terminally fused biotin ligase. Network: full STRING network; edges: confidence; active interaction sources: Textmining, experiments, databases, K-means clustering. Using STRING protein-protein interaction and functional enrichment analysis, three distinct clusters can be identified among the proteins identified using N-terminally-fused biotin ligase. C1: Proteins associated with the biological processes ribosomal RNA (rRNA) maturation, ribosomal assembly and biogenesis and the molecular functions RNA binding molecules and structural constituents of the ribosome. C2: STRIPAK complex proteins with the molecular function armadillo repeat domain and protein phosphatase 2a binding. C3: Proteins involved in mRNA maturation comprise components of the PRP19 complex and the U2-type catalytic step 2 spliceosome. C4: Proteins associated with the maturation of precursor Large SubUnit (LSU) ribosomal RNA (rRNA) molecules into mature LSU-rRNA molecules, rRNA processing and ribosome biogenesis. C4 molecular functions include RNA helicase activity, RNA binding and processing. C5: Proteins involved in mRNA splicing *via* the spliceosome and associated with the PRP19 complex and the U2-type catalytic step 2 spliceosome. **(B)** STRING protein interaction database analysis of proteins identified as MAP4K4 interactors in DAOY cells using N- or C-terminally fused biotin ligase. Analysis as in **(C)** C6: Proteins involved in glycosaminoglycan catabolism and biosynthesis, with predicted association with lysosomal and Golgi lumens, the plasma membrane, collagen-containing extracellular matrix and the cell surface. C7: Proteins associated with positive regulation of exosome assembly and secretion, regulation of neurotransmitter receptor localization and synapse assembly. C7 proteins are predicted to be associated with the plasma membrane, the cell surface, cell junctions and the lumen of intracellular organelles. C8: Proteins of the casein kinase 2 complex and of the small subunit processome, predicted implication in ribosome biogenesis, mitophagy, adherens junction regulation and PD-L1 expression in cancer. **(C)** Predicted biological processes of protein clusters identified by MAP4K4 N- and C-terminus directed proximity ligation.

Despite of this increasing list of interactors and effectors of MAP4K4, several key aspects of MAP4K4 regulation and function remain unclear. Two recent studies thus comprehensively addressed the interaction landscape of MAP4K4 to uncover regulators and targets of MAP4K4. The first study by Seo et al. (5) used a tandem affinity approach combined with mass-spectrometry, the second study by Migliavacca et al. used the BioID technology (98), where a biotin ligase was coupled either to the N- or the C-terminus of MAP4K4 and biotinylated proteins were analyzed by mass-spectrometry (62). Seo et al. compared the interactome of MAP4K4, TNIK and MINK, thereby identifying 26 MAP4K4-specific interactors in HEK293T cells that were not found in pull-downs with either TNIK or MINK (**Figure 4A**). Migliavacca et al. determined the MAP4K4 interactome in HEK293T cells and in the medulloblastoma cell line DAOY. The comparison of the interactors identified with the two different purification methods revealed that the most robust interactors of MAP4K4 are the members of the STRIPAK complex (STRN, STRN3, STRN4, STRIP1 and MOB4). In addition, eleven proteins (FGFR1OP2, SIKE1, SLMAP, RPL32, CSNK2B, TANC2, C7orf50, CACYBP, RBM34 and CCDC137) were identified as high probability interactors of MAP4K4 in HEK293T cells (**Figure 4B**). The functional significance of the interaction of MAP4K4 with these proteins remains to be determined. The Bio-ID approach identified a considerably larger number of proteins. This may be explained by the higher stringency of the tandem purification and the capability of the BioID methodology to also detect relatively weak and indirect interactors. Eleven per cent of the BioID-identified MAP4K4 interactors contain a WD40 domain (STRN, STRN3, STRN4, DCAF7, PLRG1, PPP2R2A, PRPF19, PWP1, TBL2, TBL3, WDR6 and WDR75), suggesting that this domain could mediate some of the interactions between MAP4K4 and its effectors. Among the proteins detected using MAP4K4 with N-terminally-fused biotin ligase, STRING protein-protein interaction and functional enrichment analysis identified three distinct clusters (C1-3, **Figures 5A, C**)): C1 proteins are associated with the biological processes ribosomal RNA (rRNA) maturation, ribosomal assembly and biogenesis and have molecular functions as RNA binding molecules and structural constituents of the ribosome. C2 consists of STRIPAK complex proteins with the molecular function armadillo repeat domain and protein phosphatase 2a binding. C3 proteins are involved in mRNA maturation and consist of components of the PRP19 complex and the U2-type catalytic step 2 spliceosome. Among the proteins detected using MAP4K4 with C-terminally-fused biotin ligase, STRIPAK complex proteins (C2) and two additional clusters C4 and C5 can be identified. C4 is associated with the biological processes maturation of a precursor Large Subunit (LSU) ribosomal RNA (rRNA) molecule into a mature LSU-rRNA molecule, rRNA processing and ribosome biogenesis. C4 molecular functions include RNA helicase activity, RNA binding and processing. Like C3, C5 is involved in mRNA splicing *via* the spliceosome and associated with the PRP19 complex and the U2-type catalytic step 2 spliceosome. The STRIPAK protein cluster C2 was also detected in the non-related MB cell line DAOY in proximity of MAP4K4 with N-terminally fused biotin ligase (**Figures 5B, C**). In addition, C6 proteins involved with the biological processes glycosaminoglycan catabolism and biosynthesis were detected, which are associated with the cellular components lysosomal and Golgi lumen, the plasma membrane, collagen-containing extracellular matrix and the cell surface. Among the proteins detected in DAOY cells using MAP4K4 with C-terminally-fused biotin ligase are again STRIPAK complex proteins (C2). Additionally, proteins grouped in C7 are associated with positive regulation of exosome assembly and secretion, regulation of neurotransmitter receptor localization and synapse assembly. C7 proteins are predicted to be associated with the plasma membrane, the cell surface, cell junctions and the lumen of intracellular organelles. C8 consists of proteins of the casein kinase 2 complex and of the small subunit processome, suggesting the implication of these proteins in ribosome biogenesis, mitophagy, adherens junctions regulation and PD-L1 expression in cancer.

The proteomic interaction studies hint towards functions of MAP4K4 in post-transcriptional control of gene expression through the interaction with spliceosome-associated proteins and regulators of rRNA processing and ribosomal biogenesis. The detection of syndecans, sorting nexins and caveolae-associated proteins as putative MAP4K4 interactors further indicate a regulatory role of MAP4K4 in membrane-proximal regulation of endo- and exocytotic processes. This notion is supported by a recent study on MAP4K4-dependent composition of the membrane-associated proteome, which found that MAP4K4 is indispensable both for the specific turnover as well as the maintenance of cortical proteins in response to growth factor stimulation (64).

## 2 Discussion

The potential off-target activities of pharmacological MAP4K inhibition and toxicities observed with first-generation MAP4K4 inhibitors argue for the specific repression of certain but not all effector functions of MAP4K4 to halt its oncogenic or tumor-promoting functions. Pharmacological inhibition or genetic interference with PKCθ for example, an effector kinase active downstream of MAP4K4 in invasion control (62), blocks pro-invasive signaling mediated by MAP4K4. Besides targeting of effector kinases with established inhibitors, future studies should also aim at specifically interrupting the interaction of MAP4K4 with effector proteins using small molecule compounds or therapeutic peptides. Latter strategy was successfully used to interfere with the interaction between STRN3 and PP2A, which resulted in the reactivation of STRIPAK kinases and induction of tumor-suppressive Hippo signaling (69). Depletion of STRN3 or deletion of the CNH domain of MAP4K4, which is necessary for STRN interaction, impairs pro-invasive effector functions of MAP4K4 (62). Hence, inhibition of the MAP4K4-STRN3 protein-protein interaction (PPI) by small molecules or peptides that bind to the CNH domain could yield therapeutic tools for specific repression of selected MAP4K4 functions.

The interaction proteomics indicates the regulation of RNA splicing and maturation events by MAP4K4 (clusters 3-5). This unexplored aspect of potential MAP4K4 functions will need further investigation to experimentally consolidate the current predictions. It may lead to the identification of novel MAP4K4 effector mechanisms at the level of post-transcriptional control of gene function. Posttranscriptional control such as the generation of alternatively spliced isoforms may not only contribute to tumorigenesis but also play essential roles in controlling tumor cell response to variable tissue environments during progression or treatment. Importantly, alternative splicing is a recognized process contributing to tumorigenesis and tumor maintenance, but how alternative splicing is regulated in different tumors and how alternatively spliced protein isoforms contribute to tumor onset, growth and progression remains incompletely understood (99). Notably, distinct isoforms of MAP4K4 were found to constitute a biological mechanism of MAP4K4 control in colorectal cancer, whereby isoforms 2 (NM_145686) and 5 (NM_001242560) of MAP4K4 contribute to a more aggressive phenotype (100). Alternative splicing and exon retention in *MAP4K4* was also associated with recurrent but not primary glioblastoma (101). Thus, targeting either splicing-regulatory functions of MAP4K4 or the alternatively spliced variants of MAP4K4 specifically could yield highly selective therapies for repressing the invasion-promoting functions of MAP4K4 only.

Additional indications both experimental and predicted indicate MAP4K4 involvement in endocytic processes. Initially identified as a component of integrin-containing endosomes (102), MAP4K4 was also found to promote endocytic up-take in growth factor-stimulated cells (6) and to regulate the plasma membrane-associated proteome (64). Proteomic analysis also identified proteins involved in exosome assembly and secretion as MAP4K4 interactors (62), raising the possibility that MAP4K4 promotes secretory vesicular trafficking events affecting tumor cell-host tissue interactions more broadly. An improved molecular understanding of MAP4K4 regulation of endocytic and exocytic trafficking could therefore pave ways for novel interference strategies to control MAP4K4 functions that enable surface expression of immune-modulatory and promigratory proteins such as CD155 and CD276, or regulators of ion homeostasis including solute carriers, ATPases and the chloride intracellular channel 1 (64). Latter may be of particular relevance for central nervous system malignancies, where it is overexpressed and contributes to the growth and worse outcome in pediatric medulloblastoma (103).

The repression of selected MAP4K4 activities will only be possible when reagents can be made available that specifically target the functional interaction of MAP4K4 with its effectors. Priority should thus be given to the characterization of the molecular details of such interactions, to provide the rational basis for compound design. An additional challenge is the difficulty of dissecting the relative contribution of kinase activity, of allosteric changes induced in the effector by MAP4K4 binding and of combinations of phosphorylation and binding. Hence studies that provide such information for MAP4K4 will be instrumental for rationalizing drug design and compound discovery for this fascinating kinase.

## Author contributions

All authors listed have made a substantial, direct, and intellectual contribution to the work and approved it for publication.
